# Synovial joint cavitation initiates with microcavities in interzone and is coupled to skeletal flexion and elongation in developing mouse embryo limbs

**DOI:** 10.1242/bio.059381

**Published:** 2022-06-15

**Authors:** Minwook Kim, Eiki Koyama, Cheri M. Saunders, William Querido, Nancy Pleshko, Maurizio Pacifici

**Affiliations:** 1 Translational Research Program in Pediatric Orthopaedics, Division of Orthopaedic Surgery, Department of Surgery, The Children's Hospital of Philadelphia, Philadelphia, PA 19104, USA; 2 Department of Bioengineering, Temple University, Philadelphia, PA 19122, USA

**Keywords:** Synovial joint cavitation, Microcavities, Infrared spectral imaging, Skeletal flexion and elongation, Hyaluronic acid

## Abstract

The synovial cavity and its fluid are essential for joint function and lubrication, but their developmental biology remains largely obscure. Here, we analyzed E12.5 to E18.5 mouse embryo hindlimbs and discovered that cavitation initiates around E15.0 with emergence of multiple, discrete, µm-wide tissue discontinuities we term microcavities in interzone, evolving into a single joint-wide cavity within 12 h in knees and within 72-84 h in interphalangeal joints. The microcavities were circumscribed by cells as revealed by *mTmG* imaging and exhibited a carbohydrate and protein content based on infrared spectral imaging at micro and nanoscale. Accounting for differing cavitation kinetics, we found that the growing femur and tibia anlagen progressively flexed at the knee over time, with peak angulation around E15.5 exactly when the full knee cavity consolidated; however, interphalangeal joint geometry changed minimally over time. Indeed, cavitating knee interzone cells were elongated along the flexion angle axis and displayed oblong nuclei, but these traits were marginal in interphalangeal cells. Conditional *Gdf5Cre*-driven ablation of *Has2* – responsible for production of the joint fluid component hyaluronic acid (HA) – delayed the cavitation process. Our data reveal that cavitation is a stepwise process, brought about by sequential action of microcavities, skeletal flexion and elongation, and HA accumulation.

This article has an associated First Person interview with the first author of the paper.

## Abstract (precis)

**Summary:** Synovial joints contain a fluid-filled cavity crucial for skeletal motion and lifelong function, but the developmental biology of cavitation remains largely obscure, hampering basic and translational progress.

## INTRODUCTION

The synovial joints are essential for unhindered and frictionless movement, flexion and endurance of the skeleton through life (Archer et al., 1999; Hunziker et al., 2007; Longobardi et al., 2015). They exhibit a diversity of shapes, size and organization, with each joint functionally fitted to anatomical site and the type and range of skeletal motion such as in a knee, shoulder or digits (Decker, 2017; Rux et al., 2019, 2021). Despite such diversity, the joints share basic structural components that include an insulating capsule, a lubricating fluid, ligaments and articular cartilage, all cooperating to sustain joint function and operating as an organ (Loeser et al., 2012). Each of these components as well as specialized components such as meniscus have distinct structures and phenotypes needed to exert their respective and diverse mechanical, physical and biological roles (Longobardi et al., 2015). Changes in the above basic structural and phenotypic features in joint tissues due to injury, mechanical load shifts or natural aging can derange homeostasis and lead to common joint diseases such as osteoarthritis (Sandell, 2012).

These and other aspects of synovial joint biology and pathology have been delineated over the years and are widely recognized (Longobardi et al., 2015; Pacifici and Koyama, 2021). In contrast, the developmental biology of synovial joints remains less understood, a reflection of its sheer complexity but not lack of interest (Chijimatsu and Saito, 2019; Decker, 2017; Rux et al., 2019; Salva and Merrill, 2017). Classic studies established long ago that the initial skeletal blueprint in early embryonic limbs is composed of uninterrupted and continuous cartilaginous anlagen without joints (Hamrick, 2001; Hinchliffe and Johnson, 1980). The initial recognizable morphological sign of incipient joint formation is the emergence of an interzone at each prospective joint anatomical site, separating the adjacent skeletal primordia and composed of closely juxtaposed mesenchymal cells (Haines, 1947; Holder, 1977; Mitrovic, 1978). The interzone is needed for joint formation since its microsurgical removal was shown to cause fusion of flanking skeletal elements (Holder, 1977). Relying on the fact that interzone cells express growth and differentiation factor 5 (*Gdf5*) (Storm and Kingsley, 1999), with our collaborators, we carried out genetic cell lineage tracing studies with *Gdf5-Cre* mice and found that the interzone cells and their progenies give rise almost exclusively to joint tissues, including articular cartilage, intra-articular ligaments and synovial capsule, thus representing a specialized cohort of progenitors endowed with joint tissue formation capacity (Decker et al., 2017; Koyama et al., 2008; Rountree et al., 2004). Other studies using vital dye cell tracking and inducible *Gdf5-CreER* reporter approaches showed that additional mesenchymal cells are recruited from the immediate interzone surroundings and are incorporated into joint development, generating certain portions of the joints (Hyde et al., 2008; Pacifici et al., 2006; Shwartz et al., 2016). More recent studies have focused on regulation and long-term maintenance of specific joint tissues and roles of embryonically born progenitors in adult joint tissue repair (Decker et al., 2017; Feng et al., 2019; Haseeb et al., 2021; Roelofs et al., 2017).

Despite the impressive progress outlined above, much remains unexplained and poorly understood in limb synovial joint development and morphogenesis. Notably and surprisingly, one of the least understood aspects of joint formation is cavitation, a fundamental process by which a fluid/macromolecule-filled cavity is created in the middle of the otherwise compacted interzone and will endow the joint with its essential ability to permit and sustain frictionless skeletal motion. Because cavitation creates a tissue discontinuity, early studies examined the possibility that interzone cells would undergo apoptosis or other cell death processes, in turn freeing the flanking tissues and permitting their separation (Abu-Hijleh et al., 1997; Haines, 1947). However, those findings were not confirmed by subsequent studies (Ito and Kida, 2000; Kavanagh et al., 2002). More tellingly, other studies found that cavitation in mouse and chick embryo limbs is related to local production of hyaluronic acid (HA) and expression of its cell surface receptor CD44 (Craig et al., 1990; Edwards et al., 1994; Pitsillides et al., 1995). HA-cell surface interactions in conjunction with HA water imbibition and swelling capacity (Cowman, 2017) are thought to contribute to joint cavitation by relaxation of interzone cell–cell links, decreases in matrix stiffness and build-up of fluids (Lamb et al., 2003). In line with these ideas, conditional ablation of the main HA synthase 2 (*Has2*) throughout the mouse embryo limb mesoderm using *Prx1-Cre* mice was found to delay joint cavitation though also causing considerable skeletal growth abnormalities (Matsumoto et al., 2009). There is also experimental evidence for the notion that joint cavitation is aided by muscle-driven skeletal movement. Cavitation defects were demonstrated in several joints in chick embryo limbs after *in ovo* administration of neuromuscular paralyzing drugs (Murray and Drachman, 1969; Osborne et al., 2002) and in the limbs of muscle-less mouse embryo mutants lacking muscle master genes *Myf5* and *MyoD* or *Pax3*, though knee cavitation was unaffected in these mutants (Kahn et al., 2009; Nowlan et al., 2010a). In sum, overall aspects of the cavitation process have been described. However, key details remain unclear including how the cavitation process initiates and proceeds, what the nature of incipient and developing synovial fluid may be, and to what extent the cavitation process is invariant in different limb joints. Our data provide unprecedented insights into these and related questions and in particular reveal that joint cavitation initiates with microcavities and is linked to degree of flexion and elongation of flanking skeletal elements.

## RESULTS

### Knee joint cavitation is a rapid process and starts with microcavities

**Fig. 1.**
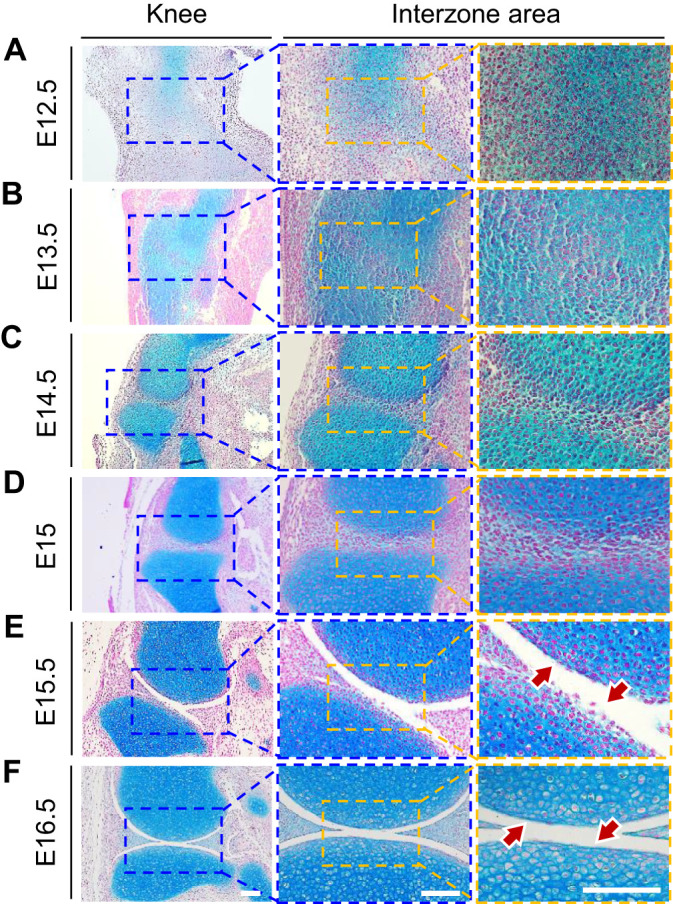
**Knee joint cavitation starts and ends within a short interval.** (A) Sections of E12.5 knee region co-stained with Nuclear Fast Red and Alcian Blue showing that the interzone was barely appreciable at this stage. Boxed area on left panel is shown at higher and higher magnification in central and right panels, respectively. (B,C) Sections from E13.5 and E14.5 knee region in which the interzone is well defined and displays typical traits including high cell density and flat cell architecture compared to flanking cartilaginous skeletal anlagen that contain round and sparser cells. (D) At E15.0, the knee interzone is still largely compacted but by E15.5 (E), the joint is essentially fully cavitated. Note that at this E15.5 stage (E), the articular surfaces encircling the synovial cavity are still rough and uneven and display irregularly shaped and roundish interzone-derived cells (arrows on right hand panel). (F) By E16.5, the articular surfaces are smooth and continuous and are lined by typical flat, elongated and seemingly contiguous cells representing the incipient surface zone of articular cartilage. Scale bar: 100 µm in all panels. *n*=3 embryos per time point.

At the outset, we carried out a systematic spatiotemporal and morphological assessment of joint cavitation in mouse embryo hindlimbs aiming to establish the time interval during which cavitation starts and ends, basic information missing in the current literature. Thus, we collected mouse embryos from E12.5 to E16.5 every 12 or 24 h (midnight and noon relative to E0.5, respectively) and processed their hindlimbs for serial paraffin sectioning along sagittal and parasagittal planes. To evaluate tissue organization and structure, the sections were stained with nuclear fast red to depict cell distribution and orientation in combination with Alcian Blue to distinguish cartilaginous skeletal elements from interzone. Staining with Hematoxylin and Eosin (H&E) was used to confirm overall tissue features. Focusing on the knee first, we found that the interzone was barely appreciable in E12.5 embryos as to be expected (Fig. 1A) (Koyama et al., 2008). It became evident at E13.5 and E14.5 and displayed typical traits such as compacted structure, high cell density and flat cellular architecture compared to the flanking cartilaginous skeletal anlagen containing round-shaped and more dispersed chondrocytes (Fig. 1B,C). The interzone was still largely compacted at E15.0 (Fig. 1D) but had essentially undergone full cavitation by E15.5 (Fig. 1E), a mere 12 h interval. At this stage, the articular surfaces encircling the incipient synovial cavity were still rough and were lined with irregularly shaped interzone-derived cells (Fig. 1E, arrows in right hand panel). The surfaces had become smooth and even by E16.5 and were lined by typically flat, elongated and seemingly contiguous cells (Fig. 1F, arrows, right hand panel).

**Fig. 2.**
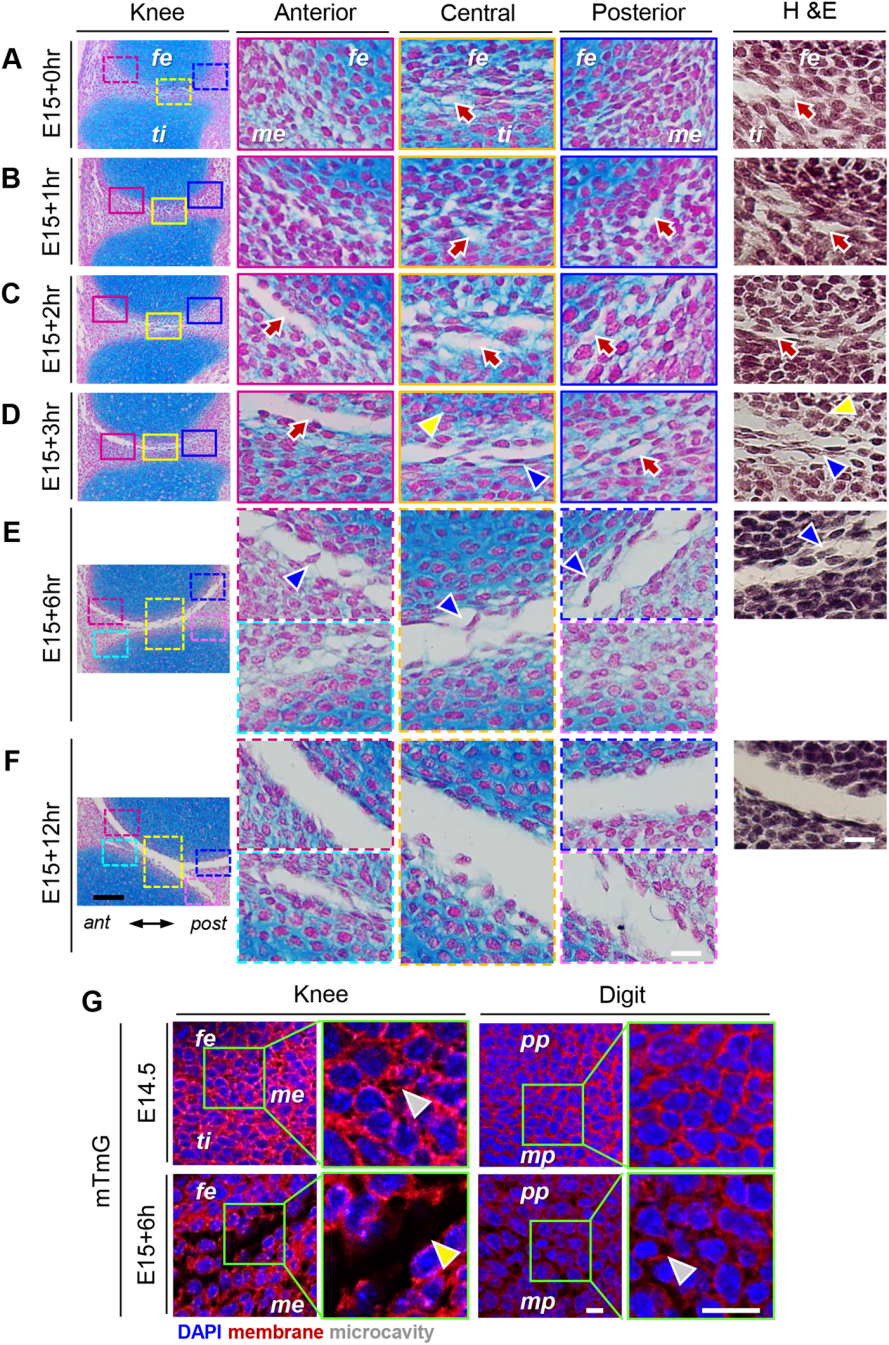
**Knee joint cavitation starts and ends within 12 h and is a stepwise process.** (A,B) Serial sections of E15.0-E15.0 plus 1 h knees stained with Nuclear Fast Red, Alcian Blue or H&E showing that the interzones are still largely compact at these stages but displays a few appreciable microcavities averaging 1-3 µm in width (arrows). (C,D) The number of discernable microcavities becomes higher at E15.0 plus 2-3 h, displaying larger and varying average sizes that may reflect expansion and/or coalescence. Note that microcavities are more conspicuous anteriorly than posteriorly (blue box areas), reflecting the overall direction of cavitation along the anterior to posterior axis. (E) By E15 plus 6 h, microcavities are largely absent within the cavitating interzone and a large fraction of the joint is occupied by a single cavity. The immature nature of the cavity is reflected by the presence of interzone-derived cells bridging the opposing and uneven articulating surfaces (red and yellow boxed areas, arrowheads). The posterior distal portion of the interzone is still largely uncavitated (pink boxed area). (F) By E15.0 plus 12 h (E15.5), much of the cavity has formed and spans the joint. (G) Confocal imaging of sections from *mTmG* mice showing that rare microcavities detectable in knee joint at E14.5 (left top panels) and the more numerous microcavities detectable at E15.0+6 h (left bottom panels) are in intimate juxtaposition with, and circumscribed by, fluorescent cells. This is more clearly appreciable at higher magnification (boxed areas, arrowheads). In developing digits (right panels), the largely uncavitated interzone at E14.5 between proximal (pp) and medial phalange (mp) displays very rare to no microcavities (right top panels). These become appreciable at E15+6 h, particularly at higher magnification (right bottom panels, arrowhead). fe, femur; ti, tibia; me, meniscus; pp, proximal phalange; mp, medial phalange. Scale bars for A-F: 100 µm, far-left panels; 20 µm, center and right panels; 10 µm, *mTmG* panels. Scale bars for G: 75 µm for low mag panels; 25 µm for high mag panels. *n*=3 embryos per time point.

The sheer rapidity of the knee cavitation process from E15.0 to E15.5 raised the question as to whether cavitation occurred suddenly and simultaneously across the joint line or might exhibit special mechanisms and features instead. Thus, we collected mouse embryos at hourly intervals from E15.0 to E15.5 and processed their hindlimbs as above. Because of the very short time frame and possible inherent developmental variability (Miyake et al., 1997), embryo collection and hindlimb analyses were performed on several independent litters and experimental repeats. We discovered that, remarkably, the interzone progressively lost its compacted and dense nature over hourly intervals and came to display an increasing number of tissue gaps and discontinuities, referred to as ‘microcavities’ in this paper, among its cells (Fig. 2). Thus, a few such microcavities were discernable in the interzone at/around E15.0 and displayed an initial average width of about 1-3 µm (Fig. 2A,B, arrows). They became more readily apparent in E15.0 plus 2-3 h (Fig. 2C,D, arrows) and displayed larger and varying average sizes, probably reflecting individual expansion and/or coalescence into each other (Fig. 2C,D). Topographically, at this stage, the microcavities were conspicuous in the interzone between femur and tibia (Fig. 2C,D, yellow boxed area) and femur and anterior meniscus (Fig. 2C,D, red boxed area) but were rare in the posterior joint region where the interzone was still largely uncavitated (Fig. 2C,D, blue boxed area). It was also apparent that many cells within the cavitating interzone, including those flanking the microcavities, often displayed a stretch-out morphology with their nucleus elongated along the antero–posterior axis (Fig. 2D, blue arrowhead). This cytoarchitecture was distinct from that of cells in neighboring cartilage that displayed round nuclei and round cell shapes and were presumably experiencing minimal to no stretch (Fig. 2D, yellow arrowhead). By E15.0 plus 6 h, the microcavities were no longer apparent as individual entities within the interzone, and much of the joint was now occupied by a single cavity (Fig. 2E), except for the posterior lower area that was still largely uncavitated (Fig. 2E, pink square box on the right). Reflecting its ongoing development, the joint-wide cavity displayed some immature traits, such as interzone-derived cells oriented across the cavity and seemingly bridging the opposing surfaces (Fig. 2E, arrowheads), and uneven articulating surfaces. By E15.0 plus 12 h (E15.5), much of the cavity had formed and was continuous and smooth-lined across the joint (Fig. 2F). We should note that microcavities were never detected in tissues away from the developing joints.

To assess more accurately the topographical relationships of microcavities to cell surfaces and borders, companion sections from the above E15.0 to E15.0 plus 12 h samples were stained with H&E (Fig. 2, far right panels). It was clear that the interzone displayed an increasing number of microcavities with increasing developmental time and the microcavities were closely juxtaposed to the stained cells. To double check this conclusion, we used the *mTmG* transgenic mouse line that expresses the *Tomato* reporter as an integral membrane protein (Muzumdar et al., 2007). Using serial sectioning and confocal fluorescence imaging, we examined the knee joints from E14.5 to E15.0 plus 6 h *mTmG* mouse embryos, stages representing onset of microcavity formation and their subsequent expansion respectively. At E14.5, the knee interzone displayed an essentially continuous membrane fluorescent signal due to its compacted nature (Fig. 2G, left top panels), with rare and small microcavities encircled by the reporter-positive cells (Fig. 2G, green boxed area). By E15.0+6 h, the larger and more numerous and variously-sized microcavities were in close contiguity with, and circumscribed by, fluorescent cells (Fig. 2G, left bottom panels, arrowhead).

### Interphalangeal joint cavitation proceeds very slowly

**Fig. 3.**
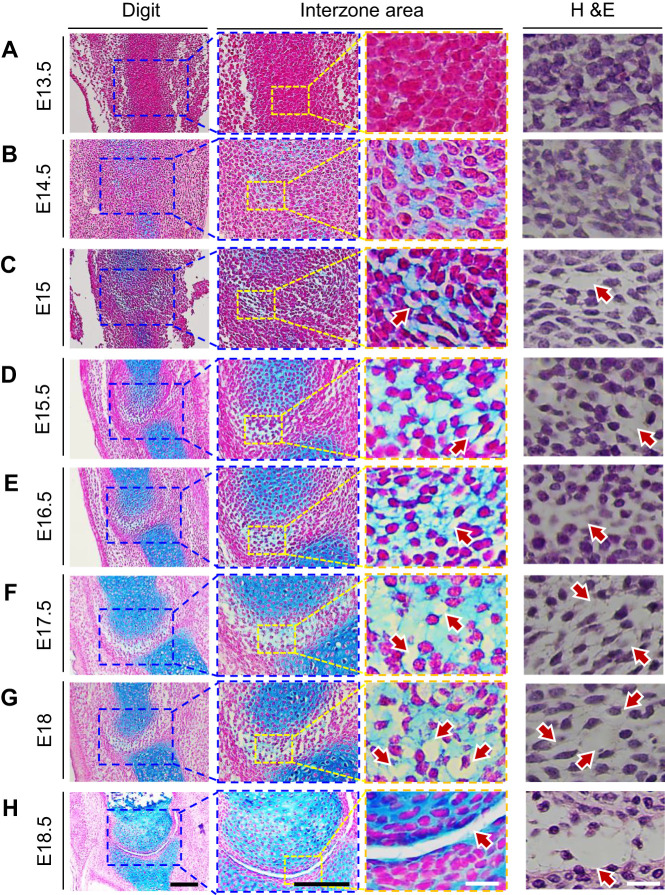
**Interphalangeal joint cavitation proceeds slowly.** (A) Sections processed for histochemical and H&E staining as above show that the metatarsal-phalangeal interzone is not yet appreciable at E13.5, but (B,C) becomes better defined by E14.5-E15.0. (D-G) At subsequent stages and up to E18.0, the interzone becomes more and more evident but does not undergo full cavitation, unlike knee's interzone that is fully cavitated by E15.5. however, the interphalangeal interzone does display microcavities that increase in number over time (D-G, arrows). Note that at all stages examined, the cells retain a roundish architecture and do not appear to be elongated or stretched in any particular direction, reflected also by the round shape of their nuclei. (H) By E18.5, a joint-wide cavity has formed, particularly in the anterior portion of the joint. Scale bars: 100 µm for left panels (black color) and 20 µm for central and right panels (white color). *n*=3 embryos per time point.

Next, we asked whether cavitation in distal limb joints involved similar kinetics, spatiotemporal processes and timing as seen in the knee. Thus, we examined serial sagittal and parasagittal sections of metatarsal–phalangeal joints from E13.5 to E18.5 mouse embryos after histochemical staining of cell surface and matrix components as above. The interzone was not yet appreciable at E13.5 (Fig. 3A) but was well defined by E14.5-E15.0 (Fig. 3B,C). At variance with knee's interzones at those stages, the interphalangeal cells were largely round and did not appear to be elongated in any particular direction. Thereafter, the interzone began to lose its compact structure and exhibited an increasing number of microcavities randomly dispersed along the putative joint line (Fig. 3D-G). Analysis of *mTmG* specimens at E14.5 and E15+6 h showed that the emerging microcavities were fully encircled by cells as seen in knee (Fig. 2G, right panels). This phase proceeded slowly and lasted until E18.0, and it was not until E18.5 when a single joint-wide cavity had formed, though the opposing articulating surfaces were still rough and uneven (Fig. 3H). Thus, the cavitation process in the autopod region also involves microcavities as a morphological starting point but is much slower and seemingly exposed to lower strain than in the knee.

### Infrared spectral imaging reveals microcavity content

**Fig. 4.**
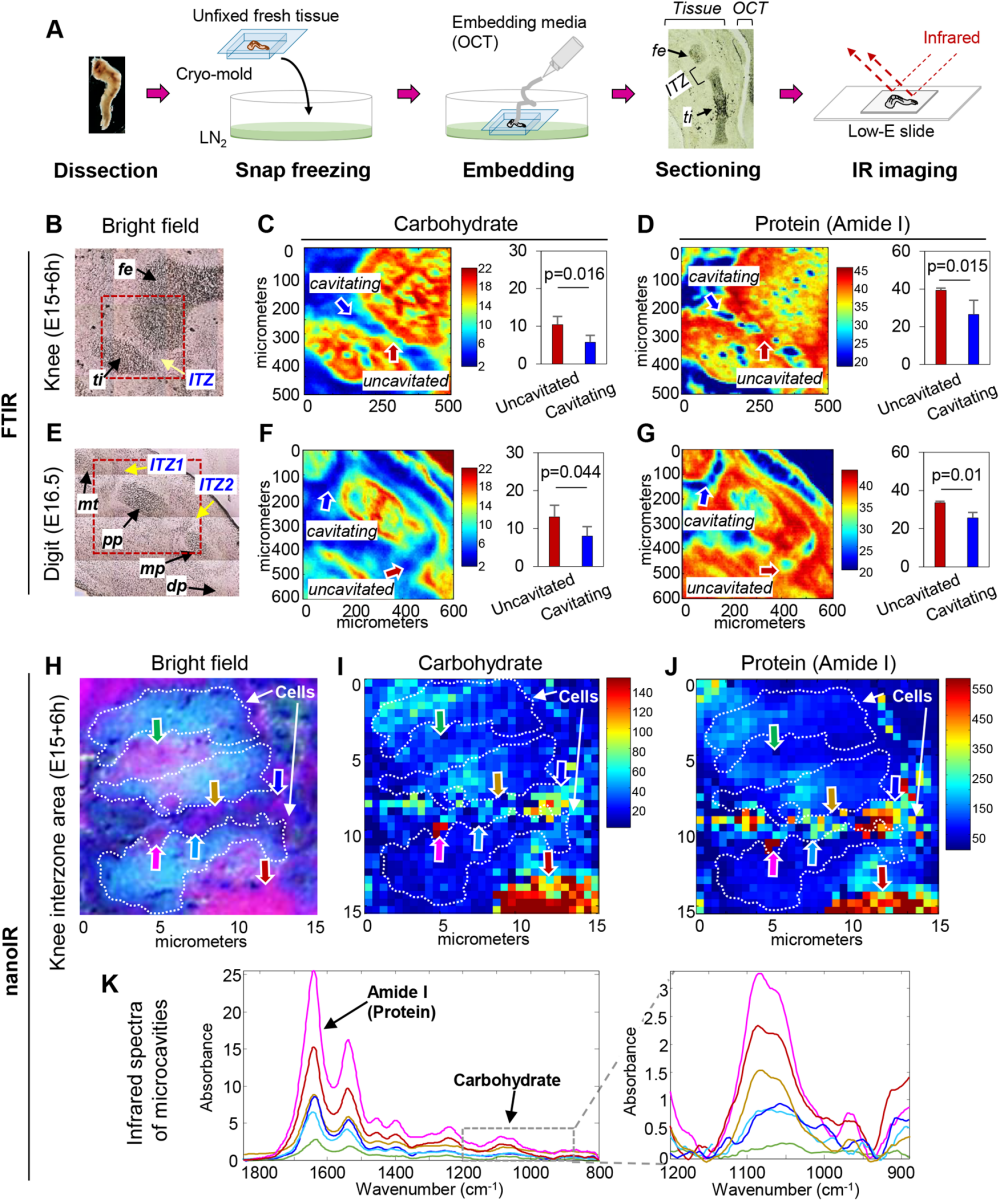
**Infrared spectral imaging reveals microcavity content.** (A) Schematic diagrams of sample preparation and data acquisition using Infrared spectral imaging. (B,C) Histological bright field and FTIR images of E15.0+6 h knee area of interest (B, box) and respective 1140-980 cm^−1^ range spectrum showing that carbohydrate levels are conspicuous in cartilaginous anlagen reflective of their high glycosaminoglycan content, but not so in cavitating interzone. With this specimen, the anterior cavitated portion of interzone (blue arrow) has a carbohydrate content about 45% lower than in the posterior largely intact portion (red arrow; *P*=0.016). (D) Amide I band spectrum analysis (1720-1592 cm^−1^ range) indicating that total protein levels are about 30% lower after cavitation than prior to (*P*=0.015). (E-G) Analogous FTIR analyses carried out in digit joints show that carbohydrate and protein levels are lower in proximal cavitating tarsal–first phalangeal joint interzone than in developmentally younger and largely uncavitated distal interzone between first and second phalange anlagen (38% lower carbohydrate, *P*=0.044; 24% lower protein, *P*=0.01). (H-J) Bright field and nano-IR imaging spectroscopy images from representative area within cavitating E15.0 plus 6 h knee interzone rich in microcavities. Cell bodies are delineated by dotted contours. Nano-IR imaging of individual sites (colored arrows) indicate variability in carbohydrate and protein content levels amongst microcavity. (K) Infrared spectra of individual sites (shown in corresponding color) clearly indicate that microcavity content of protein and carbohydrates varies quantitatively. (*n*=4 per group, *P*<0.05). fe, femur; ti, tibia; mt, metatarsal; pp, proximal phalange; mp, medial phalange; dp, distal phalange.

If the microcavities represent a precursor of, and a step toward, full joint cavitation, they should be filled with incipient components and water eventually leading to development of a functional synovial fluid containing hyaluronate, Prg4 and phospholipid lubricants (Kosinska et al., 2012; Levick and McDonald, 1995). Thus, what could be the microcavities’ initial content and how could it be examined? To tackle this challenging question, we resorted to infrared spectral imaging that allows detection and analysis of tissue molecular and macromolecular components at µm spatial resolution, using protocols long established in our labs (Boskey and Pleshko Camacho, 2007; Kim et al., 2005; Querido et al., 2021). Accordingly, E15.0-E16.5 mouse embryo hindlimbs were snap-frozen in liquid nitrogen to keep tissue components as intact as possible and were quickly embedded in optimal cutting temperature (OCT) compound, preventing infiltration of embedding medium that could cause spectral interference (Fig. 4A). We first used a Spotlight Fourier transform infrared (FTIR) imaging spectrometer with a 6.25 µm pixel resolution and focused on the delineation of carbohydrate and protein content within cavitating interzones and surrounding tissues. In E15.0 plus 6 h knees, imaging in the integrated area of absorbance band at 1140-980 cm^−1^ spectrum showed that carbohydrate levels were extremely high within the cartilaginous anlagen as to be expected for being rich in glycosaminoglycans (GAGs), but levels were considerably lower within cavitating interzone (Fig. 4B,C). In this specimen, the anterior portion of the interzone was fully cavitated and displayed a carbohydrate content (sugar band; 1140-980 cm^−1^) appreciably lower than that of the posterior and largely uncavitated region (about 45% lower; *P*=0.016) (Fig. 4C). When we measured total protein content (amide I band; 1720-1592 cm^−1^), we found that protein levels were also lower after than prior to cavitation (32% less; *P*=0.015) (Fig. 4D).

We extended these analyses to E16.5 autopod joints and observed that carbohydrate and protein levels were also lower in fully cavitated proximal interzones between tarsal and first phalange anlagen than in developmentally younger and largely uncavitated distal interzones between first and second phalange anlagen (38% lower carbohydrate, *P*=0.044; 24% lower protein, *P*=0.01) (Fig. 4E,G).

Microcavities were difficult to analyze specifically using the above standard pixel resolution. Thus, we extended our analyses by using submicron optical photothermal infrared (O-PTIR) spectral imaging (nano-IR) instrumentation that can acquire data at 0.5 µm spatial resolution. Within E15.0 plus 6 h knees, we selected representative regions of the interzone undergoing cavitation and zoomed into areas rich in microcavities. Bright field images produced by this instrumentation allowed us to distinguish cells appearing in light blue color (Fig. 4H, dotted outlines) from extracellular entities and materials, including interzone-associated microcavities, appearing in shades of purple (Fig. 4H). Nano-IR imaging indicated that there was variability in content levels amongst interzone microcavities as revealed by signal intensity at each 0.5 µm spatial resolution spot (Fig. 4I,J, arrows). Spectra derived from each site indicated that the microcavity content included protein and carbohydrates, again eliciting varying signal intensities (Fig. 4K). In general, overall protein and carbohydrate levels per unit area were lower in microcavities than neighboring tissues such as cartilage, likely reflecting accrual and build-up of water, dilution of macromolecular content and establishment of an incipient synovial fluid.

### Knee but not digit joints exhibit increasing flexion and angulation over time

**Fig. 5.**
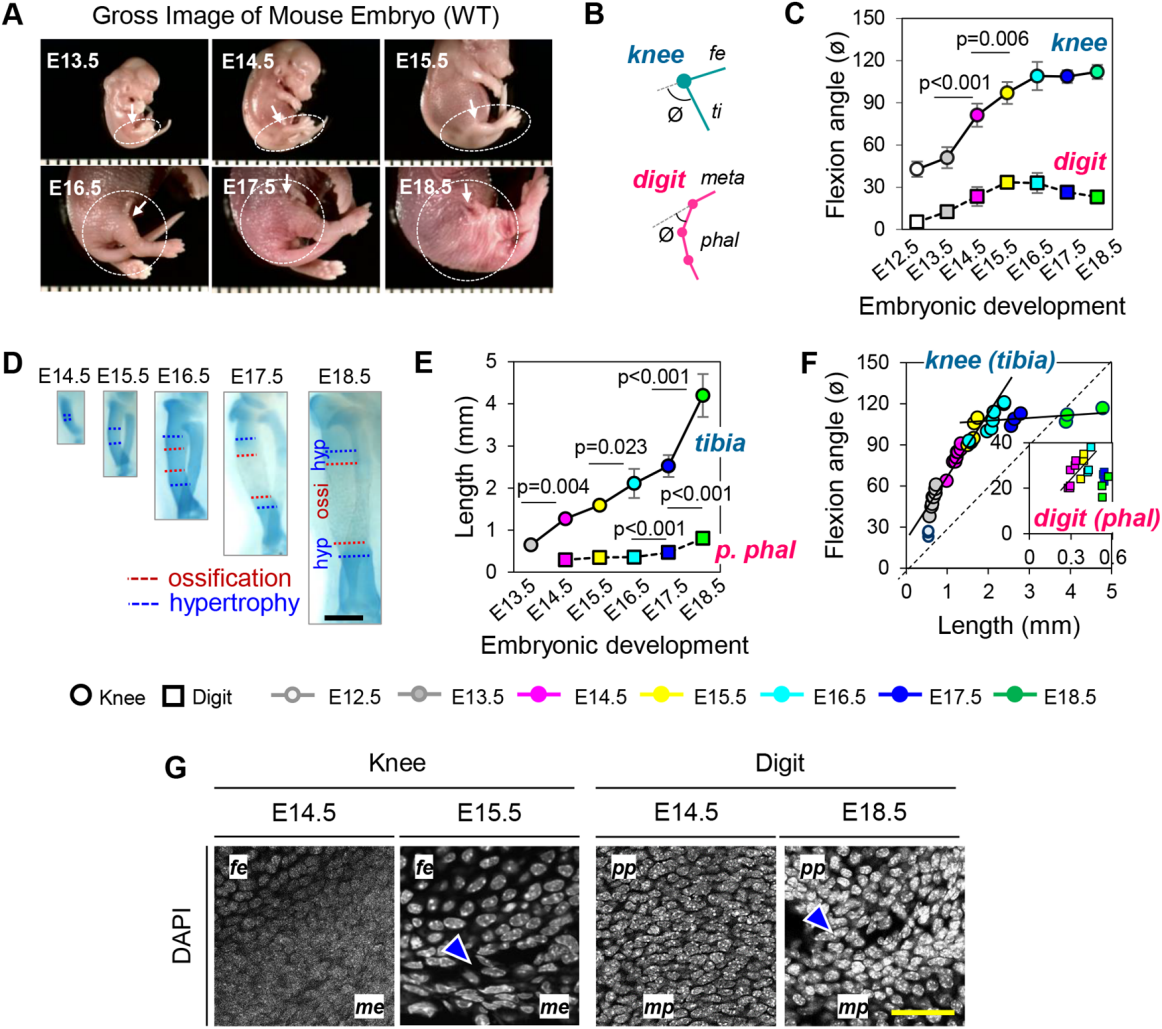
**Knee but not digit joint exhibit increasing flexion and angulation over time.** (A) Photo images of freshly harvested wild-type mouse embryos from E13.5 to E18.5. Hindlimb and knee region are encircled. (B) Schematic describing flexion angle (ø) measured in knee and digit joint. (C) Graphs relating flexion angle to embryonic age and showing that knee flexion angle increases significantly from approximately 50^◦^ at E13.5 to about 80^◦^ at E14.5 (*P*<0.001 versus E13.5); about 97^◦^ by E15.5 (*P*=0.006 versus E14.5); and a maximum of 110^◦^ at E16.5, plateauing thereafter (circles). In contrast, digit joint angulation is minimal and slightly increases to about 34^◦^ by E15.5, plateauing thereafter (squares). (D) Images of representative Alcian-Blue-stained tibiae from E14.5 to E18.5 used to monitor lengthening over time (scale bar: 500 µm). (E) Graphs depicting tibia and proximal phalangeal lengths measured on Alcian-Blue-stained specimens over time. Data indicate tibia anlage lengthening from approximately 0.65 mm at E13.5 to: about 1.26 mm at E14.5 (*P*=0.004 versus E13.5); about 2.1 mm by E16.5 (*P*=0.023 versus E14.5); and 4.2 mm by E18.5 (*P*<0.001 versus E16.5). In comparison, proximal phalanges grow moderately from about 0.29-0.36 mm at E14.5-E16.5 to and about 0.47 mm at E17.5 (*P*<0.001 versus previous stages) and 0.8 mm by E18.5 (*P*<0.001 versus E17.5). (F) Calculated relationships between joint flexion angle and skeletal element length indicating that ratios for knee increase significantly up to E16.5 (slope=1.6), whereas the ratios for phalangeal joint is largely linear and exhibits a minor increase, eliciting a slope=1.1 (circles=knee, squares=digit). (G) Nuclear DAPI staining indicating that interzone cells engaged in knee cavitation at E15.5 display elongated nuclei along the antero-posterior axis (arrowhead), but nuclear shape remains largely round in E14.5 knee interzone cells and in E14.5 and E18.8 phalangeal cells. Scale bar: 50 µm; *n*=4-10 per group per time point.

The differences in cavitation timing, rates and patterns in developing knee versus digit joints above were major and significant, but what could be their origin? In carrying out our studies, we had noted that in freshly isolated embryos, the knee joint displayed increasing angulation between femur and tibia with increasing developmental age (Fig. 5A, arrows), but this change appeared to be minor in autopod joints over time. Suspecting that such differences in angulation degrees could have mechanistic significance for cavitation rates, we sought to analyze and quantify it. Hindlimbs from E12.5 to E18.5 mouse embryos were collected at 12 and 24 h intervals and carefully processed for whole mount staining with Alcian Blue (Fig. S1). Their hindlimbs were inspected for overall skeletal and joint development and for assessment and measurement of joint flexion angle between femur and tibia and between metatarsal and first phalangeal elements (Fig. 5B,C). Knee flexion angle (ø) was approximately 50° at E13.5, increased to about 80° at E14.5 (*P*<0.001 versus E13.5), further increased to about 97^◦^ by E15.5 (*P*=0.006 versus E14.5) and reached a maximum of 110° at E16.5, plateauing thereafter (Fig. 5C, circles). In sharp contrast, digit joint angulation was nearly absent up to around E14.5 and slightly increased to a maximum of about 34° by E15.5, remaining at about that level thereafter (Fig. 5C, squares). Thus, the increasing and eventually steep flexion angle in developing knees was directly related to their rapid rates of cavitation.

What could drive flexion and angulation? An obvious possibility is that skeletal muscles were involved but knee cavitation was not affected in muscle-less mouse embryo mutants (Kahn et al., 2009; Nowlan et al., 2010a). An alternative possibility is that the increasing flexion angle in knees was driven by proximo-distal elongation of flanking skeletal elements. Angulation would be needed to accommodate the fast-elongating elements experiencing physical resistance from surrounding tissues and ligaments. Thus, we measured the approximate lengths of tibias as well as proximal phalanges by microscopic quantification in hindlimbs over developmental time. The recognizable tibia anlagen at E13.5 were approx. 0.65 mm long, increased to about 1.26 mm at E14.5 (*P*=0.004 versus E13.5) and further grew to about 2.1 mm by E16.5 (*P*=0.023 versus E14.5) and 4.2 mm by E18.5 (*P*<0.001 versus E16.5) (Fig. 5D,E). In comparison, Alcian-Blue-stained proximal phalanges remained within a range of 0.29-0.36 mm long from E14.5 to E16.5 and then increased to about 0.47 mm at E17.5 (*P*<0.001 versus previous stages) and 0.8 mm by E18.5; *P*<0.001 versus E17.5) (Fig. 5E). Thus, we calculated the relation between joint flexion angle and skeletal element length and found that for the knee, the ratio sharply increased until E16.5 (slope=1.6) and then plateaued. In contrast, the ratio for phalangeal joint was largely linear and exhibited a minor increase, eliciting a slope=1.1 (Fig. 5F).

The sharp differences in flexion angle and rates in knee versus phalangeal joints above should have elicited different degrees of antero–posterior strain on local cells. As a measure of that possibility, we examined more closely the morphology and shape of the nuclei which are known to reflect directional physical forces experienced by cells (Szczesny and Mauck, 2017). Nuclear staining with DAPI showed that interzone cells in pre-cavitation E14.5 knee mostly displayed a uniformly round nucleus (Fig. 5G, far left panel), but those engaged in cavitation at E15.5 did display elongated nuclei along the antero–posterior axis (Fig. 5G, left center panel). For comparison, interzone cell nuclei in phalangeal joints did not exhibit notable changes over developmental time and maintained a round and seemingly relaxed architecture (Fig. 5G, right hand panels). These data conform to those stemming from histological and histochemical analyses shown in Figs 2 and 3.

### Cavitation is delayed by conditional ablation of *Has2*

**Fig. 6.**
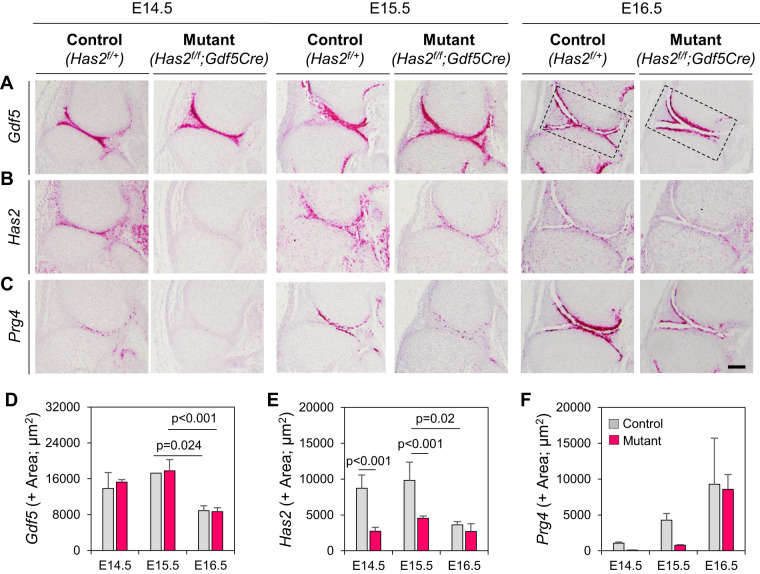
**Expression patterns of key joint genes diverge over time.** High resolution RNAscope hybridization was used to reveal patterns. (A) Stereotypic interzone marker gene *Gdf5* is strongly expressed prior, during and after cavitation in control E14.5, E15.5 and E16.5 knees, with a slight decrease at the latter stage. *Gdf5* expression is not appreciably affected after conditional deletion of *Has2* via *Gdf5Cre*. (B) The major joint lubricant-encoding gene *Has2* is also strongly expressed in E14.5 and E15.5 control knees. The gene was effectively ablated in conditional *Has2^f/f^;Gdf5Cre* mouse embryo knees at all stages examined. (C) The second major lubricant-encoding gene *Prg4* is instead barely expressed in control E14.5 and E15.5 knees but become conspicuously expressed by E1.6.5. Such expression patterns are negatively impacted by conditional *Has2* ablation. (D-F) Quantitative analyses of above gene expression patterns in control and conditional *Has2* mutant knees using ImageJ. Statistical significance observed between pairs are specified within the graphs. Scale bar: 50 µm; *n*=3 per group per time point.

The carbohydrate content detected by IR within the microcavities is likely to represent HA for the most part. This possibility is in line with several past studies showing that cavitating interzones become rich in HA and the local cells express the HA cell surface receptor CD44 (Craig et al., 1990; Edwards et al., 1994; Pitsillides et al., 1995). In addition, *Has2* ablation throughout the early limb mesoderm via *Prx1Cre* or *HoxB6Cre* was shown to alter the cavitation process and reorient it spatially, respectively (Liu et al., 2013; Matsumoto et al., 2009). Because those approaches did not target the joints specifically and caused widespread changes on the overall process of limb skeletogenesis, it remains unclear whether *Has2* has a specific and local role in interzone functioning and development. Thus, we first reassessed *Has2* expression during joint development using RNAscope and found that it was significantly expressed in, and restricted to, the interzone already in the largely uncavitated E14.5 knees (Fig. 6B). *Has2* remained strongly expressed at E15.5 but appeared to be decreased by E16.5 (Fig. 6B,E; *P*<0.02). *Has2* expression patterns closely paralleled those of the stereotypic interzone marker *Gdf5* (Fig. 6A,D). Interestingly however, expression of another major joint lubricant, Prg4/lubricin, was barely appreciable at E14.5 and E15.5 but became stronger by E16.5 (Fig. 6C,F), indicating that it does not have a significant role in the onset of cavitation.

**Fig. 7.**
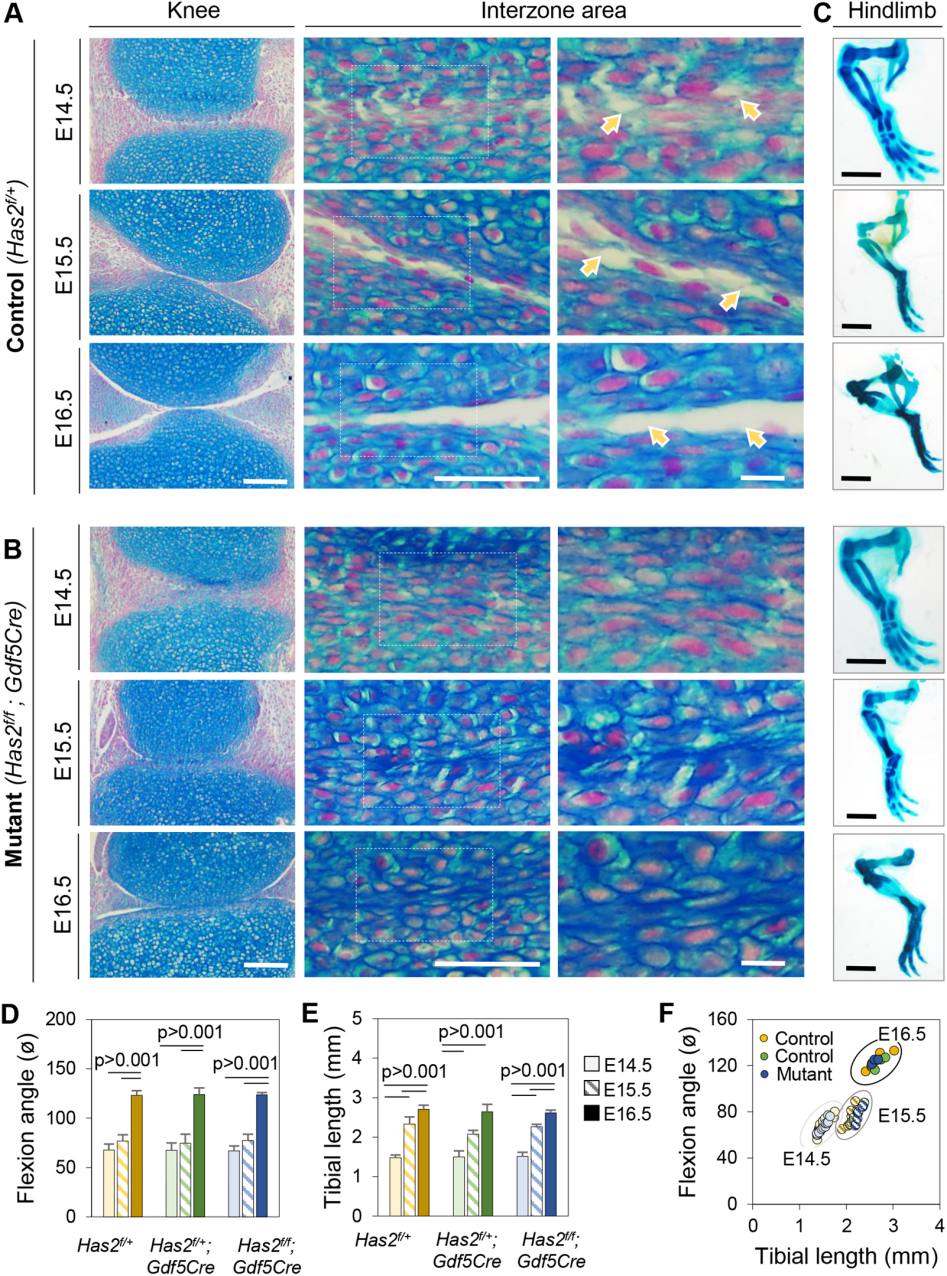
**Cavitation is delayed by conditional joint-specific ablation of *Has2*.** (A,B) Images of Alcian-Blue-stained sections of E14.5 to E16.5 knees showing typical developmental steps in cavitation in control *Has2^f/+^* mouse embryos including microcavity formation (indicated by arrows in panel A). Those steps are clearly delayed in companion conditional *Has2^f/f^;Gdf5Cre* mutants (B). (C) Global view of whole-mount Alcian-Blue-stained hindlimbs from control and mutant embryos at similar E14.5 to E16.5 stages showing that overall development and flexion of limbs was not affected. (D,E) Measurements of flexion angle and average tibia length indicating that these parameters are invariant in controls versus mutants. (E) Correlation plots flexion angle and tibia length showing lack of changes in mutants (*n*=4 per group per time point). Scale bars for B: 100 µm for left column, 50 µm for middle column; 10 µm for right column; scale bar for C: 1 mm.

To test whether *Has2* has a specific role in cavitation and joint development, we created conditional *Has2* mutants by mating floxed *Has2* mice (*Has2^f/f^*) with *Gdf5Cre* mice and examined the joint phenotypes over time, using heterozygous *Has2^f/+^;Gdf5Cre* or *Has2^f/+^* mice as controls. Histochemical staining showed that cavitation was markedly delayed in conditional homozygous mutants as shown by reductions in microcavity number and joint-wide cavity in E14.5 through E16.5 knees (Fig. 7B) compared to companion controls (Fig. 7A). RNAscope confirmed that ablation of *Has2* was considerable though not complete and the mutants expressed much lower levels of this gene compared to controls (Fig. 6B; *P*<0.001). Interestingly, *Gdf5* expression remained strong and seemingly unaffected in the mutants (Fig. 6A) but *Prg4* was still minimally expressed by E16.5 (Fig. 6C), indicating that the overall cavitation process and cavity phenotypic maturation were delayed in the *Has2* mutants. Lastly, we measured skeletal flexion and elongation given their apparent roles in cavitation and joint development. However, we found no appreciable differences in these parameters as well as in the ratios between the parameters (Fig. 7C-F) indicating that cavitation had been delayed in the mutants but preserved enough to permit skeletal flexion and elongation over time.

## DISCUSSION

Our data provide previously unsuspected insights into the processes that lead the compacted mesenchymal interzone to undergo cavitation, along the path to forming a fluid-filled, frictionless synovial cavity. Rather than occurring suddenly and widely, the cavitation process in mouse embryo hindlimbs proceeds in morphologically distinct and local steps, made readily apparent by the approaches used here. As early as E14.5, the interzone begins to loosen its notoriously-compacted, high-cell-density organization, in which the cells are initially linked to one another intimately and directly (Archer et al., 1994; Mitrovic, 1978). Progressively separated cells (referred to here as microcavities), are shown by *mTmG* imaging to be closely surrounded by the cells and shown by IR spectroscopy to exhibit low, appreciable amounts of protein and carbohydrate content, with bound water likely to be present throughout. The microcavities are clearly restricted to a prescribed joint line within the middle of the interzone, are absent in neighboring tissues, increase in number and average size over time, and are eventually replaced by a single, joint-wide cavity. Though our static approaches do not allow us to monitor microcavity evolution over time by live microscopy or other means, the data do suggest that the microcavities would merge into each other over time, with this activity being important, perhaps essential, in the eventual creation of a single, joint-wide cavity. Because cavitation proceeds anteriorly to posteriorly over time, it is possible that the microcavities may also allow for finer spatiotemporal control and local modulation of the process.

The interzone is not a mere signpost indicating where a joint will form, and its cells do not simply die out and disappear to make room for a cavity to form. Rather, the cells persist and actively participate in the formation of distinct joint tissues, serving as unique joint tissue progenitors (Decker et al., 2017; Koyama et al., 2008; Shwartz et al., 2016). It is this realization that has generated much interest in understanding how a cavity could form within the interzone, splitting it approximately into proximal and distal halves that in turn continue to function and generate the opposing articulating tissues and surfaces separated by the cavity. Past studies cited above have postulated that local production and accumulation of HA would have an important role in loosening interzone cell-cell links, attracting water by imbibition and gradually separating the cells along the joint line (Craig et al., 1990; Edwards et al., 1994; Pitsillides et al., 1995). Those studies raise the possibility that the carbohydrate content we detect in microcavities by IR imaging might largely if not exclusively represent HA. Those studies and their implications are also very much in line with our new findings here that joint-specific conditional ablation of *Has2* markedly delays cavitation, manifested by concurrent reductions in microcavity number, distance of opposing articulating surfaces, and cavity volume over time. In addition, other studies have demonstrated the expression in cavitating interzones of proteases such as Adamts-1, proposed to cleave local matrix components including versican, reduce cell–pericellular-matrix interactions and propel cavitation forward (Capehart, 2010; Nagchowdhuri et al., 2012). Though details remain unclear, those studies and our data here suggest that onset and progression of cavitation rely on the following spatiotemporal series of biological events: loosening of interzone cell–cell and cell–matrix interactions by proteases and HA production; creation, enlargement and merging of microcavities; attraction and buildup of fluid (water); and final establishment of a single, joint-wide cavity.

Skeletogenesis does not obey biological rules and mechanisms only but is also closely dependent and reliant on mechanical cues and forces (Henderson and Carter, 2002; Nowlan et al., 2014; Shea et al., 2015). Cavitation and joint formation do conform to this trend, and studies dating back decades have focused on the roles of muscle-driven motion on joint development and cavitation (Mitrovic, 1982) as well as on long term joint maintenance and endurance (Eckstein et al., 2006). Mutant muscleless mouse embryos do display a severe developmental retardation and even fusion of certain joints, such as elbow and shoulder, but the developing knee is not affected and cavitates and develops normally (Kahn et al., 2009; Nowlan et al., 2010a,b). Such diverse consequences on joint formation in muscleless mutants have remained unexplained and puzzling ever since they were first reported, but our data here finally provide an explanation based on skeletal flexion and elongation occurring over embryonic age. We find that knees start and complete cavitation in a mere 12 h period and their flanking skeletal elements flex and elongate considerably, whereas the interphalangeal joints take over 72 h to cavitate and their elements flex and grow moderately. Thus, there appears to be a direct proportionality – and perhaps a causal link – between cavitation and skeletal flexion/elongation. Intriguingly, Nowlan et al. measured the length of forelimb and hindlimb skeletal elements in control and muscleless mutants and found that mutant humerus and ulna anlagen were shorter, whereas mutant tibia was of normal length compared to controls (Nowlan et al., 2010a). Thus, elbow fusion in those mutants could be due in part or largely to reduced mechanical inputs and forces from the shorter flanking skeletal elements. Knees in the mutants would cavitate normally because tibia elongates normally and would exert a normal degree of mechanical force. While these theses need to be tested directly, they underline the basic notion that close interplay and coordination between biological and mechanical mechanisms control the spatiotemporal progression of skeletogenesis and joint cavitation and formation along the limbs. Possible roles of joint ligaments would need to be considered and tested in the future since these structures could influence extent, directionality and impact of mechanical forces within developing joint tissues.

The synovial fluid is well recognized for its key role in protecting joints from physical abrasion and maintaining frictionless joint motion and endurance. The fluid is water based and its main lubricans are HA, Prg4/lubricin and phospholipids (Blewis et al., 2007). Its functional importance is demonstrated by the fact that alterations in fluid volume and/or composition are linked to joint diseases including OA (Catterali et al., 2010). These and other basic aspects of synovial fluid biology are well recognized, but underlying mechanisms regulating its homeostasis are less understood, including how its volume is maintained and whether production of different lubricans is coordinated. Our data provide some insights into these basic questions. We find that *Has2* is already expressed along, and restricted to, the future joint site even before the onset of cavitation becomes morphologically recognizable. *Has2* continues to be locally expressed as cavitation advances, being then shifted to the opposing articulating surfaces upon completion. In comparison, *Prg4* expression remains barely detectable before and during cavitation and increases appreciably only after cavitation is completed. The data reaffirm the notion that *Has2*-produced HA is important for cavitation to begin, possibly loosening interzone cell–cell links and attracting water into the microcavities. Prg4/lubricin would have little if any roles in these initial key steps, as also indicated by the fact that conditional *Prg4* ablation does not affect cavitation, though joint function is hampered postnatally (Rhee et al., 2005). Interestingly, the delay in *Prg4* expression we observed in the conditional *Has2* mutants indicates that *Prg4* expression is secondary to -and dependent on- cavity establishment, possibly triggered by HA-driven fluid build-up and hydrostatic pressure and/or greater skeletal motion permitted by the newly established cavity. These interplays between *Has2* and *Prg4* expression could operate in adult joints as well to modulate lubricant production and/or composition as needed. There is also evidence that HA fragments are present in synovial fluid in adult joints (Tamer, 2013) and that further HA processing by HYAL1 is needed for joint endurance (Higuchi et al., 2017). HA fragments are known to have distinct biological activities as shown in other systems (Cyphert et al., 2015), and it will be of interest to determine whether and when they may occur during joint development and cavitation and what function they may exert, including on *Prg4* expression.

## MATERIALS AND METHODS

### Mouse strains, mating and genotyping

All animal procedures were approved by the Children's Hospital of Philadelphia IACUC. Wild-type CD-1 mice were from Charles River Lab, Wilmington, MA, USA; Rosa26-*mTomato/mGFP* (*R26-mTmG*) reporter mice (stock number 7676) were from Jackson Lab, Bar Harbor, ME, USA; and the *Gdf5Cre* transgenic mice (Rountree et al., 2004) and floxed *Has2* (*Has2^f/f^*) mice (Matsumoto et al., 2009) were described previously. Conditional *Has2*-deficient mice in joint-specific regions were created by mating *Has2^f/f^* mice with *Gdf5Cre* mice. For controlled timed pregnancy, mice were mated starting at 18:00 h and separated at 09:00 h. Noon on the day of separation was considered E0.5 (Decker et al., 2017). This timeline assumes mice to become pregnant around midnight, and this is generally accepted based on mouse behavior and life cycle. To address possible variability, mice used in the study were from independent litters and independent experiments.

Genotyping was carried out with DNA isolated from tail clips (Koyama et al., 2008).

### Spatiotemporal and histological analyses of cavitation

Hindlimbs were collected from E11.5 to E18.5 mouse embryos at 12- or 24-h intervals. This spectrum of embryonic ages encompasses the developmental stages prior to, during, and well after cavitation, based on previous studies. To capture knee cavitation occurring between E15 and E15.5, embryos were collected every hour during this 12 h interval. Because of inherent variability from embryo to embryo during this short interval, we collected multiple litters at each stage and carried out multiple independent experiments to verify consistency of observations at those hourly stages. Hindlimbs were fixed in 4% paraformaldehyde (PFA) for 24 h, embedded in paraffin, sectioned at 6 µm, and stained with Alcian Blue pH 2.5 and Nuclear Fast Red to depict and analyze tissues and matrices spatio-temporally during the cavitation process, focusing on the medial side of the joints. We used Alcian Blue at pH 2.5 rather than at the more stringent and common pH 1.0 to reveal both high and low anionic macromolecules including glycosaminoglycans and glycoproteins (Terry et al., 2000). Staining of sections with H&E was carried out by standard procedures (Koyama et al., 2008). Bright-field images were captured using an Eclipse Ci Nikon microscope (Nikon Instruments Inc, Melville, NY, USA) with NIS-Element software.

### Confocal imaging

Hindlimbs from *R26-mTmG* mice embryos were collected at E14.5 (before) and E15.5 (during) cavitation, fixed in 4% PFA, embedded in OCT compound, cryosectioned at 10 µm and imaged using confocal microscopy (Leica DMi8, Leica Microsystems, Germany). Cell nuclei were stained with DAPI (ThermoFisher Scientific) and cover-slipped with ProLong Gold anti-fade reagent without DAPI (Invitrogen).

### Infrared spectral imaging

Freshly dissected hindlimbs from E15.0-E16.5 mouse embryos were washed in PBS and placed on a cryomold (Sakura Finetek, Torrance, CA, USA). Excess liquid on tissue surface was removed by lightly touching with Kimwipes. Specimens in the cryomold were snap-frozen by slightly immersing them in liquid nitrogen. After placing them to dry ice, the specimens were quickly embedded in OCT compound, minimizing the possibility that embedding medium would infiltrate the tissues and cause spectral interference. Embedded samples were cryo-sectioned at 10 µm on sagittal and parasagittal planes and mounted on a Low-E slide (Kevely Technologies, Chesterland, OH, USA). The resulting unfixed and unstained cryo-sections were dried at room temperature overnight. They were scanned the following day using a Spectrum Spotlight 400 FTIR spectrometer (Perkin-Elmer, Waltham, MA, USA) (Boskey and Pleshko Camacho, 2007) initially at standard 6.25 µm spatial resolution and 8 cm^−1^ spectral resolution. To further investigate tissue and microcavity content, submicron resolution scale O-PTIR spectral imaging (nanoIR) was carried out at 0.5 µm spatial resolution and 2 cm^−1^ spectral resolution using a mIRage IR Microscope (Photothermal Spectroscopy Corp, Santa Barbara, CA, USA). Acquired data were processed and analyzed using ISys software 5.0 (Malvern Instruments Ltd., Worcestershire, UK). The integrated area of the absorbance band at 1140-980 cm^−1^ (C-O-C and C-OH ring vibrations) was used to characterize carbohydrate content (Boskey and Pleshko Camacho, 2007; Kim et al., 2005). The integrated area of the absorbance band at 1720-1592 cm^−1^ (amide I; C=O stretch) was used to map protein content. A minimum of three samples per group/stage were used for quantitative analyses.

### Whole-mount embryo staining and skeletal anlage analyses

Hindlimbs from E11.5 to E18.5 mouse embryos were collected every 24 h (*n*=5 per group) and processed for whole-mount embryo staining with Alcian Blue (Rigueur and Lyons, 2014). After staining, samples were transferred to 1% potassium hydroxide and glycerol mixture in a series of mixture ratios to hydrolyze soft tissues, leading to transparency and visualization of Alcian Blue-stained skeletal elements. Under a Nikon SMZ-U stereo/dissecting microscope equipped with a SPOT Insight 2MP CCD color digital camera (SPOT Imaging, Sterling Heights, MI, USA), stained hindlimbs were dissected out and images were captured on sagittal and coronal axes using SPOT imaging software (SPOT imaging, Sterling Heights, MI, USA). Images were used to calculate the length of skeletal elements and flexion angle (ø) of knee and digit (metatarsal–phalangeal) joints by ImageJ software.

### *In situ* hybridization

RNAscope *in situ* hybridization was carried out using RNAscope2.5 HD Detection reagent-RED (Advanced Cell Diagnostics, Newark, CA, USA) to determine the spatio-temporal expression patterns of *Gdf5* (catalogue number 407211), *Has2* (catalogue number 465171) and *Prg4* (catalogue number 437661) in developing knee joints. Briefly, after paraffin removal and rehydration, serial tissue sections were pretreated with a custom reagent and hybridized with each probe for 2 h at 40°C in a custom oven. Signal was amplified with a pre-amplifier and multiple amplifiers as per the manufacturer's protocols. The final hybridization signal was detected and visualized by reaction with Fast Red substrate for 10 min at room temperature. Companion sections were hybridized with positive (catalogue number 313911) or negative control probes (catalogue number 310043) to assure signal specificity. Sections were counterstained with hematoxylin, dried and cover-slipped.

### Statistical analyses

Statistical analyses were conducted using the SYSTAT software (version 13, SYSTAT software Inc., San Josh, CA, USA). Significance was determined by two-way ANOVA with Tukey's *post hoc* test (*P*<005).

## Supplementary Material

Supplementary information
