# Supplementary figures and images for: Synovial joint cavitation initiates with microcavities in interzone and is coupled to skeletal flexion and elongation in developing mouse embryo limbs
Source: Biol Open. 2022 Jun 15;11(6):bio059381. doi: 10.1242/bio.059381 (PMC9212078; doi:10.1242/bio.059381)

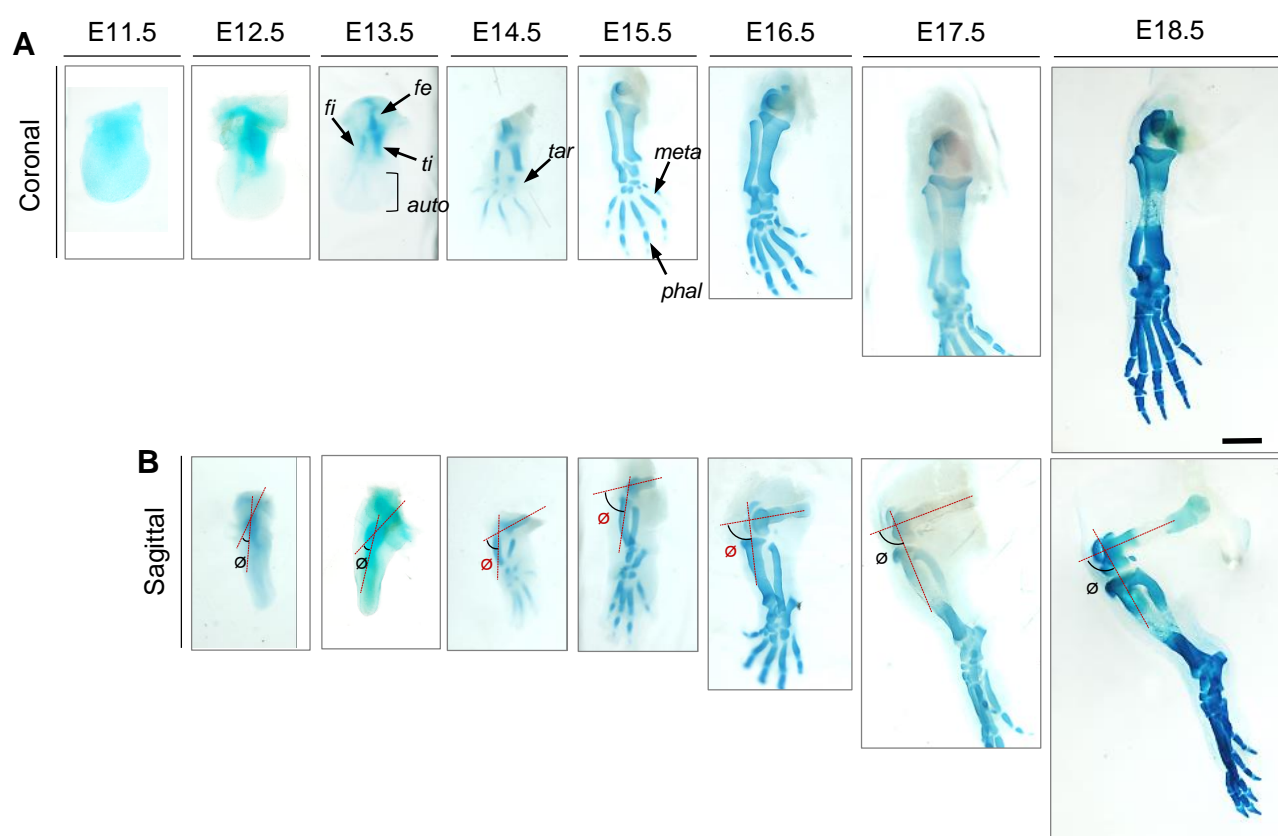

**Fig. S1.** Flexion angle ( $\varnothing$ ) and Skeletal growth

Supplement: Supplementary information [file biolopen-11-059381-s1.pdf]
